# Supplementary material for: The impact of biological sex in peripheral nerve blockade: A prospective pharmacodynamic, pharmacokinetic and morphometric study in volunteers
Source: PLoS One. 2024 Jan 26;19(1):e0297095. doi: 10.1371/journal.pone.0297095 (PMC10817111; doi:10.1371/journal.pone.0297095)
Supplement: S1 Protocol — (DOCX) [file pone.0297095.s002.docx]

Medical University of Vienna
Spitalgasse 23
A-1090 Vienna

The Impact of Biological Gender in Peripheral Nerve Blockade: A Prospective Study in Volunteers

CLINICAL STUDY PROTOCOL, Version 1.1

SHORT TITLE: Gender_PNB

Date: 20.11.2022

**Principal Investigator according to**

**Austrian drug law (AMG §§ 2a, 35, 36)**: Daniela Marhofer^1^, MD

**Sponsor (AMG §§ 2a, 31, 32):** Medical University of Vienna

**Monitor (AMG §§ 2a, 33, 34)** Gerhard Garhöfer^2^, MD

**Study nurse:** Maria Weber^2^

**Statistical Expert:** Malachy Columb^3^, MD

**Protocol Authors (in alphabetic order):** Malachy Columb^3^, MD
 Daniela Marhofer^1^, MD
 Peter Marhofer^1,4^, MD
 Philipp Opfermann^1^, MD
 Werner Schmid^1^, MD
 Thomas Stimpfl^5^, PhD
 Markus Zadrazil^1^, MD
 Markus Zeitlinger^2^, MD

^1^ Department of Anaesthesia, General Intensive Care Medicine and Pain Therapy, Medical University Vienna, Vienna, Austria

^2^ Department of Clinical Pharmacology, Medical University Vienna, Vienna, Austria

^3^ Manchester University Hospitals NHS Foundation Trust, Wythenshawe Hospital, Manchester, UK

^4^ Department of Anaesthesia, Orthopedic Hospital Speising

^5^ Clinical Department of Laboratory Medicine, Medical University of Vienna, Vienna, Austria

TABLE OF CONTENTS

[Synopsis 7](#_Toc152685055)

[Introduction 12](#_Toc152685056)

[SCIENTIFIC / MEDICAL SECTION 14](#_Toc152685057)

[Scientific background 14](#_Toc152685058)

[Study groups and nerve block technique 14](#_Toc152685059)

[Sequence of Nerve Blockades 15](#_Toc152685060)

[Dosing rationale 15](#_Toc152685061)

[Assessment of sensory and motor blockade 15](#_Toc152685062)

[Needle Material 16](#_Toc152685063)

[Sterility 16](#_Toc152685064)

[Nerve Measurements and Evaluation of Nerve Visibility^1^ 16](#_Toc152685065)

[Pharmacokinetic Analysis 17](#_Toc152685066)

[Objectives of the study 18](#_Toc152685067)

[Primary Outcome Parameter 18](#_Toc152685068)

[Secondary Outcome Parameters 18](#_Toc152685069)

[Null-hypothesis and alternative-hypothesis 18](#_Toc152685070)

[Study design 18](#_Toc152685071)

[Study population 19](#_Toc152685072)

[Inclusion criteria for healthy volunteers 19](#_Toc152685073)

[Exclusion criteria for healthy volunteers 19](#_Toc152685074)

[Reason for withdrawal 20](#_Toc152685075)

[Study drug 21](#_Toc152685076)

[Ropivacaine 21](#_Toc152685077)

[Pharmacokinetic data 21](#_Toc152685078)

[Complete description of indications, contraindications, undesirable and general side effects 21](#_Toc152685079)

[Study protocol 22](#_Toc152685080)

[Screening visit (day -14 to day-1) 22](#_Toc152685081)

[Study day 22](#_Toc152685082)

[Final examination 22](#_Toc152685083)

[METHODS 23](#_Toc152685084)

[Nerve blocks 23](#_Toc152685085)

[Sensory assessment of blockade 23](#_Toc152685086)

[Motor assessment of blockade 23](#_Toc152685087)

[Definition of onset times 23](#_Toc152685088)

[Sensory blockade 23](#_Toc152685089)

[Motor blockade 23](#_Toc152685090)

[Definition of duration of blockade 23](#_Toc152685091)

[Sensory blockade 23](#_Toc152685092)

[Motor blockade 23](#_Toc152685093)

[Time points 23](#_Toc152685094)

[Blinding 23](#_Toc152685095)

[Power Analysis 23](#_Toc152685096)

[Statistical Analysis 24](#_Toc152685097)

[Drug Storage 25](#_Toc152685098)

[Labelling 26](#_Toc152685099)

[Subject identification 26](#_Toc152685100)

[Life style restrictions 27](#_Toc152685101)

[Documentation 28](#_Toc152685102)

[Adverse events 29](#_Toc152685103)

[Definition 29](#_Toc152685104)

[Complications of Ultrasound Guided Peripheral Nerve Blocks 29](#_Toc152685105)

[Documentation and Reporting of Adverse Events 29](#_Toc152685106)

[Serious adverse events (SAEs) 29](#_Toc152685107)

[Causality Assessment between Adverse Events and study medication 30](#_Toc152685108)

[Severity of the adverse event 30](#_Toc152685109)

[Information of the regulatory authorities and the IRB-IEC 30](#_Toc152685110)

[Premature termination of the trial 31](#_Toc152685111)

[REMOVAL OF SUBJECT FROM TRIAL 32](#_Toc152685112)

[STATISTICAL / ANALYSIS SECTION 33](#_Toc152685113)

[Sample size calculation 33](#_Toc152685114)

[Interim-Analysis 33](#_Toc152685115)

[Randomization 33](#_Toc152685116)

[PD, PK and morphometric data analysis 33](#_Toc152685117)

[ETHICAL SECTION 34](#_Toc152685118)

[Safety precautions 34](#_Toc152685119)

[Inconveniences and risks for subjects 34](#_Toc152685120)

[Risk/benefit assessment 34](#_Toc152685121)

[Individual benefit 34](#_Toc152685122)

[Adverse events 34](#_Toc152685123)

[Serious adverse events 34](#_Toc152685124)

[Insurance 35](#_Toc152685125)

[Ethical and legal aspects 36](#_Toc152685126)

[Audit and Inspection 37](#_Toc152685127)

[ADMINISTRATIVE SECTION 38](#_Toc152685128)

[Publication of study results 38](#_Toc152685129)

[Protocol amendment 38](#_Toc152685130)

[Monitoring 38](#_Toc152685131)

[FUNDING 39](#_Toc152685132)

[REFERENCES 40](#_Toc152685133)

[Appendices 42](#_Toc152685134)

GLOSSARY

AE Adverse event
AUC Area under the Curve
BMI Body mass index
BP Blood pressure
BW Body weight
C_max_ Maximum drug concentration
CRF Case Report Form
CSA Cross Sectional Area
ECG Electrocardiogram
GCP Good Clinical Practice
HR Heart rate
IMP Investigational medicinal product
IP In-plane needle guidance technique
IRB-IEC Institutional Review Board – Independent Ethics Committee
i.v. Intravenous
JPEG Joint Photography Expert Group
NSAID Non-Steroidal-Anti-Inflammatory-Drug
OTC Over the counter
PD Pharmacodynamic
PK Pharmacokinetic
PTT Partial thromboplastin time
RBC Red blood count
SUSAR Suspected Unexpected Serious Adverse Reaction
t_1/2_ Termination elimination half-time
t_max_ Time to maximum concentration
WBC White blood count

# Synopsis

| TITLE | **The Impact of Biological Gender in Peripheral Nerve Blockade: A Prospective Study in Volunteers** |
| --- | --- |
| Phase | IV |
| INVESTIGATIONAL PRODUCTS | **Drug:** Ropivacaine  ***Concentration:*** 7.5 mg/mL (0.75%) |
| BACKGROUND | To evaluate PD, PK and nerve-related morphometric differences of biological gender (male versus female) on nerve block characteristics |
| OBJECTIVES | **Primary Objective**   - Duration of sensory nerve blockade   **Secondary Objective**   - Pharmacokinetic characteristics (C_max_, t_max_, t_1/2_, AUC_0-6_, AUC0-∞) - Duration of motor blockade - Onset time of sensory nerve blockade - Onset time of motor blockade - Diameter of the ulnar nerve (absolute and relative to the BMI) - Visibility of the ulnar nerve in ultrasound (Grey-tone analysis ulnar nerve relative to the surrounding tissue) |
| DESIGN | Prospective, single-centre, interim-analysis after 2 x 12 cases |
| HYPOTHESIS | Null-hypothesis: biological gender (male or women) does not influence the primary outcome variable  Alterative-hypothesis: biological gender (male or women) influences the primary outcome variable |
| CONTRIBUTION TO THE CLINICAL PRACTICE | In the case of rejection of the null-hypothesis, peripheral regional anaesthesia techniques can be adapted to the particular biological gender and PD characteristics can be adapted to the respective clinical indication. |
| BLINDING-PROTOCOL | - |
| CENTER(S) / COUNTRY(IES) | 1 centre in Austria (Department of Clinical Pharmacology, Medical University of Vienna) |
| PATIENTS / GROUPS | 45 healthy male and 45 healthy female volunteers (total number of volunteers: 90). An interim-analysis will be after 12 male and 12 female volunteers. |
| INCLUSION CRITERIA | - Healthy males and females - Healthy, according to the medical history, ECG, vital signs, laboratory results and physical examination as determined by the Investigator/Sub-Investigator - Signed written informed consent prior to inclusion in the study - 18-55 years old inclusive - BMI: 18 to 35 kg/m^2^ - Ability to understand the full nature and purpose of the study, including possible risks and side effects - Ability to co-operate with the investigator and to comply with the requirements of the entire study - Availability to volunteer for the entire study duration and willing to adhere to all protocol requirements |
| EXCLUSION CRITERIA | - Any clinically relevant abnormalities at ECG (12 leads) - Any clinically relevant abnormal physical findings - Any clinically relevant abnormal laboratory values indicative of physical illness - Ascertained or presumptive hypersensitivity to the active principle and/or formulations ingredients of the study drug - History of anaphylaxis to drugs or allergic reactions in general, which the investigator considers may affect the outcome of the study - Relevant history of malignancy, of renal, hepatic, cardiovascular, respiratory, gastrointestinal, musculoskeletal, skin (particularly at the site of drug application), haematological, endocrine or neurological diseases that may interfere with the aim of the study - Pregnancy - Any psychiatric illnesses - Any anticoagulatory or pain-therapy-related drugs (e.g. NSAIDs, opioids) from one week before until the end of the study - Using other medications during 1 week before the start of IMP application including OTC - Participation in another clinical study investigating another IMP within 1 month prior to screening - Blood donations during 4 weeks prior to this study - History of drug or alcohol abuse - Other objections to study participation in the opinion of the investigator |
| STUDY PROCEDURES | A screening visit precedes the first study day. Each volunteer will undergo one ulnar nerve blockade. For each individual, duration of the whole study (including screening and end-of-study visits) will be two weeks. The duration of the active study participation is estimated between 8 and 12 hours.  Once inclusion criteria are fulfilled and written informed consent to study participation is provided, participants will be asked to report at the study centre on the study day at a convened time.  Volunteers receive one ulnar nerve block (description of standardized performance of blockade see below) on the non-dominant side according to one of the two study groups. Evaluation of sensory blockade will be performed via Pinprick testing (method and timing of evaluation see below). Evaluation of motor blockade will be performed via the evaluation of thumb adduction (method and timing of evaluation see below).  Venepuncture blood draws will be performed throughout a vascular access for evaluation of pharmacodynamic characteristics.  Ultrasonographic appearance (visibility) of the ulnar nerve will be detected via grey scale analysis. |
| ADMINISTRATION OF STUDY DRUGS, STUDY GROUPS | **Study group M:** **M**ale volunteers receive 3 mL Ropivacaine 0.75% (7.5 mg/mL = 22.5 mg/mL) perineural to the ulnar nerve  **Study group F:** **F**emale volunteers receive 3 mL Ropivacaine 0.75% (7.5 mg/mL = 22.5 mg/mL) perineural to the ulnar nerve  Ulnar nerve blocks will be performed under sterile conditions via ultrasound guidance at the middle third of the forearm (exact definition see below). |
| ASSESSMENT OF BLOCKADE | Sensory blockade will be evaluated via Pinprick testing (0 = no sensory feeling, 100 = no difference in sensory feeling to the corresponding non-blocked side) at the hypothenar area compared with the corresponding non-blocked side.  Motor blockade will be evaluated via adduction of the thumb:  3 = no difference, adduction against contra-force possible  2 = slight difference, adduction against slight contra-force hardly possible  1 = significant difference, adduction without contra-force hardly possible  0 = no active adduction possible, paralysis |
| PHARMACOKINETIC PARAMETER | - Maximum concentration (Cmax) - Time to maximum concentration (tmax) - Terminal elimination half-life (t1⁄2) - Area under the concentration–time curve (AUC) for the sampling period of 6 h (AUC0-6) - The total AUC (AUC0-∞). |
| ULTRASOUND VISIBILITY | Difference of grey scale analysis between the median grey scale of the cross-sectional area of the ulnar nerve and areas of a similar ulnar nerve size in a 0, 3, 6 and 9 o´clock position  $Grey Scale Value Ulnar Nerve/\frac{Grey Scale Values 0,3,6,9 o´clock}{4}$  Vienna Scoring System:   1. Internal Structure of the nerve visualized 2. The nerve is visualized as a circular or oval-shaped blight halo (epineurium) 3. The nerve is visualized as reflection determined by the anatomy of the surrounded tissue 4. The anatomical position of the nerve shows no response to the ultrasound beam (isoechoic behaviour) |
| HOUSING | On study days, subjects will be confined at the clinic until the percentage of Pinprick testing is back to 20 and motor blockade back to 1. For the whole duration of their stay, they will be under constant medical supervision. |
| POWER ANALYSIS | A previous study with ropivacaine 0.75% for ulnar nerve blockade showed a duration of sensory blockade of 350 min with the largest SD of 54 minutes.^2^ We expect a true difference of approximately 35 minutes (10%). Assuming a true difference in means between the study groups of 35 minutes, a pooled standard deviation of 54 minutes, the study would require a sample size of 38 for each group (i.e. a total sample size of 76, assuming equal group sizes), to achieve a power of 80% and a level of significance of 5%. For nonparametric analysis, the sample size is increased by 15% to *90* subjects.  After 2 x 12 cases, we perform an interim-analysis to re-evaluate the number of initially calculated subjects. A subsequent power-analysis will be performed to determine the definitive number of study cases. We intend to achieve a power of 80% and a level of significance of 5% to find a nominal difference of 10 to 15%.  Interim analyses and sample size calculations will be conducted using the O’Brien-Fleming alpha spending function for test boundaries in PASS and NCSS software (NCSS Inc., Kaysville UT.) |
| STATISTICAL METHODOLOGY | Pharmacodynamic data analysis: The primary outcome is duration of sensory block. Secondary outcomes include onset of sensory block, onset and duration of motor block.  Data will be presented as mean (SD), median [interquartiles] and count (%) and analysed using unpaired two-sided Student *t-*, Mann-Whitney *U-*, log-rank and Fisher exact statistics. The primary outcome will be analysed using unpaired Student *t-*, Mann-Whitney *U-* or log-rank statistics as appropriate. Secondary analyses will include mixed effects linear models to adjust for subject and baseline characteristics and for repeated measures in subjects as random intercepts.  Pharmacokinetic data analysis: Secondary outcomes, including parameters such as C_max_, t_max_and AUC will be estimated and analysed using statistics as listed above.  Morphometric data analysis: Differences between grey scales of nerves and nerve visibility (differences between nerves and surrounding tissue) and differences in the Vienna Scoring System will be compared using unpaired two-sided Student *t-* or Mann-Whitney *U-* statistics as appropriate. Mixed effects linear models will be performed to determine the correlation of nerve visibility with morphometric variables. |

# Introduction

The importance of regional anaesthesia in perioperative medicine is increasing. Regional anaesthesia (with or without sedation / general anaesthesia) is the method of choice regarding optimized pain therapy, decreases or even avoids the quantitative demand of perioperative opioids, and is associated with non-pain related effects. Improved postoperative gastrointestinal function, decreased need of postoperative mechanical ventilation, less postoperative nausea and vomiting or less postoperative confusion are only a few non-pain related effects of regional anaesthesia.

Regional anaesthesia is in principle subclassified in central and peripheral regional techniques. Central regional anaesthesia is the umbrella term for neuraxial regional techniques, where local anaesthetics are administered in the epidural (epidural anaesthesia) or subarachnoid (spinal anaesthesia) space. Peripheral regional anaesthesia is the technical term for blockade techniques of single nerves or complex nerval structures (e.g. the brachial plexus). Ultrasonographic guidance enables a precise administration of local anaesthetic drugs around nerve structures (extra-epineural), which is associated with an excellent margin of safety (= avoidance of intraneuronal or intravascular puncture) and improved pharmacodynamic nerve-block characteristics (= improved sensory and motor nerve-blocks as compared with other techniques of nerve detection).

In 1993, Kapral et al. identified the use of ultrasound for regional anaesthesia purposes, where nerve structures, adjacent anatomical structures, the cannula and the spread of local anaesthetic solutions could be directly visualized. With the further development of ultrasound technology and an increased knowledge ultrasound guided regional anaesthetic techniques, success rates and the usage in the daily clinical practice increased worldwide. Nowadays challenges in the context of regional anaesthesia are the facts that no new specific drugs are developed and the deficiency of personalized methods. The clinical practice is mainly based on a weight dependent dosage of local anaesthetic drugs without any biological gender related considerations.

In other medical specialities (e.g. cardiology, oncology, infectious diseases), there is a worldwide trend towards personalized medicine.^3–6^ For regional anaesthesia purposes, only a few studies investigate with controversial results the possible impact of biological gender differences.^7–12^ Most of these studies did not primarily investigate the impact of biological gender in this context.

In principle, there are three different methods possible with regard to specific studies in the field of regional anaesthesia: patient-, volunteer- or animal- based studies. Our scientific study group (“Regional Anaesthesia and Paediatric Anaesthesia” at the Medical University of Vienna) has extensive experience in the field of volunteer studies.^2,13–16^ Volunteer studies in the field of peripheral regional anaesthesia are associated with the following advantages as compared with clinical studies in patients:

- The entire study period is organized and performed in an environment which is specially designed for volunteer studies.
- The daily practice in a clinical environment (e.g., operation theatre, recovery room, emergency room, etc.) is busy and the particular focus is not on data collection for studies.
- In particular in the field of regional anaesthesia, duration of sensory and motor blocks is difficult to measure due to postoperative swelling, casts, wound-dressings etc.
- Volunteers are more motivated to provide exact data regarding various study related scoring systems as comparted with patients, who are (understandably) more focused on their particular disease / injury.

We therefore designed this prospective, single-centre, volunteer-based study to investigate the impact of biological gender (male versus women) on peripheral nerve blockade. The duration of sensory block is defined as primary outcome parameter. After 2 x 12 cases, we perform an interim analysis.

# SCIENTIFIC / MEDICAL SECTION

## Scientific background

Personalized medicine is an important development in the individual treatment of patients and is based on individual and tailored medical decisions, practices and interventions. The terms personalized medicine, precision medicine and stratified medicine can be used interchangeably in this context.

Regional anaesthesia plays an important role in the context of perioperative medicine, thus providing the highest level of pain therapy during and after surgery. The use of ultrasound for the detection of peripheral nerves, the adjacent anatomy, the cannula and the spread of local anaesthetic solutions increased the efficacy of peripheral regional block techniques significantly and contributed to the reliability of these techniques. Despite a large body of evidence regarding ultrasound guided regional anaesthesia and improved regional block characteristics,^17^ there is limited knowledge in the field of personalized medicine in this context available.

A recent study illustrates gender differences in the CSA of cervical nerves with precise methods of measurements.^18^ A clinical study by Tang et al. shows prolonged blockade of the sciatic nerve in women with pre-existing diabetes.^11^ Unfortunately the authors did not measure the CSAs of individual nerves at the puncture site, but we speculate that the CSAs of nerves could correlate with the duration of sensory and motor blocks. Gender-dependent differences in the electrical conduction system of peripheral nerves could also influence the PD characteristics of regional blocks. Fujumaki et al. shows in this context a greater sensory nerve action potential of upper limb nerves in healthy females as compared with healthy men.^19^ Interestingly, these differences were only shown in upper, but not in lower limb nerves.

Conversely, Cullion et al. did not find differences of PD characteristics of nerve blocks with bupivacaine in rats. They showed a duration of sensory blockade of 158.5 [139-190] minutes (median [interquartile range]) (males) compared to 173 [134-171] minutes (females) (p = 0.702) and a duration of motor blockade of 157 [141-171] minutes (males) compared to 172 [146-320] minutes (females) (p = 0.2786). A study by Gaudreault et al. investigated the relationship between PK parameters and nerve block characteristics, and did also not find biological gender related differences.

As described above, the limited number of studies in the field of regional anaesthesia and biological gender shows controversial results. Until today, no study investigates gender-specific PD and PK characteristics of peripheral nerve blocks. We therefore designed this prospective, single-centre study in volunteers to investigate the influence of biological gender (male and women) on ultrasound guided peripheral nerve blockade.

## Study groups and nerve block technique

In the present study, 90 healthy male and female volunteers will receive ultrasound guided ulnar nerve blockade. Ultrasound guidance is the golden standard in regional anaesthetic nerve blocks with high success rates and low complications in experienced hands.

Ulnar nerve blockade: The ulnar nerve can be visualized below the level of the sulcus of the ulnar nerve and proximal where the ulnar artery joins the nerve, between the *flexor carpi ulnaris, humeroulnar head of the superficial flexor digitorum and profound flexor digitorum muscles*. The ulnar nerve shows a constant sensory distribution pattern with the lowest intra- and interindividual variability compared with other sensory- and motor nerves supplying the hand.^20^ The following combination of drugs will be administered:

*Study group M:* Ropivacaine 0.75% (Volume 3.0mL = 22.5mg) will be administered perineural to the ulnar nerve

*Study group F:* Ropivacaine 0.75% (Volume 3.0mL = 22.5mg) will be administered perineural to the ulnar nerve

### Sequence of Nerve Blockades

On the study day, blockade of the ulnar nerve with the above-described drug will be performed.

### Dosing rationale

Three ml local anaesthetic is a larger volume as described in the literature to provide an ED95 for peripheral single nerve blockade.^21^ Therefore, a reliable complete blockade can be assumed with the dose which is used in this study. We decided to administer a pre-defined volume of LA to obtain comparable PK data.

## Assessment of sensory and motor blockade

*Sensory blockade* will be assessed via Pinprick testing (100 = no difference to the contralateral side, 0 = no sensory reception) at the hypothenar area in comparison with the contralateral side. Five areas of sensory supply are defined:

- dorsal side hypothenar muscles
- ulnar side hypothenar area
- palmar side hypothenar muscles
- fifth finger
- ulnar side fourth finger.

Short bevel needles are used for Pinprick testing. The tip of the needle will be applied with a force adequate to indent the skin without puncturing it, and this will produce a consistent painful sensation when applied to non-blocked areas. This kind of sensory testing is well described in the literature.^2,13^ Evaluation of skin temperature (which is also a factor of skin-sensitivity) will not be performed because of conflicting results in the literature and various factors influencing the measurements.^22^ In addition, the sympathetic portion of the ulnar nerve is unpredictable (different to e.g. the sciatic nerve, where a large portion of sympathetic fibres can be assumed).^23^

*Motor blockade* will be assessed via adduction of the thumb:

- 3 = no difference, adduction against contra-force possible
- 2 = slight difference, adduction against slight contra-force hardly possible
- 1 = significant difference, adduction without contra-force hardly possible
- 0 = no active adduction possible, paralysis

Sensory and motor testing will be performed at the following time points (min):

Baseline, 2, 4, 6, 8, 10, 15, 20, 30, 60 min after the block, and then every 30 min until Pinprick testing = 80 and motor-scale = 1.

*Onset of sensory and motor block* is defined as Pinprick = 0 in the hypothenar area (4 from 5 above-described areas to consider anatomical variations) and motor scale = 0.

Duration of sensory and motor block is defined as Pinprick testing = 80 in the hypothenar area and motor scale = 1.

We decided to define sensory block duration when Pinprick testing = 80 (and not back to 100) to avoid an extreme long duration of investigation. According to previous results with ropivacaine 0.75% (7.5 mg/ml), the duration of complete recovery from sensory block can be estimated with > 6 hours.

### Needle Material

22G 50mm Facette tip needles with an injection line (Polymedic™, te me na, Carrières sur Seine, France) will be used for nerve blockade.

### Sterility

The puncture area will be prepared in a surgical sterile manner. The ultrasound probe will be covered with a sterile ultrasound probe cover (SaferSonic Inc., Ybbs, Austria). Sterile ultrasound gel will be used as contact medium between the ultrasound probe and the skin (SaferGel, Ybbs, Austria).

## Nerve Measurements and Evaluation of Nerve Visibility^1^

Prior nerve blockade, the ulnar nerve will be scanned in the position as described above. For all scans, a high-end ultrasound device with a 15-6 MHz linear ultrasound probe will be used (SonoSite X-Port, SonoSite Inc., Bothell, WA, USA). The nerve will be traced until optimal visibility is obtained and the brightness will be standardized using the “Autogain” setting of the ultrasound machine. Since the ultrasound visibility of a nerve is a factor of differences in brightness between the tissues, the overall gain does not influence this proportion. Thereafter the nerve circumference will be measured with the internal area measurement tool.

Figures will be stored as JPEG files with the ulnar nerve in the centre of the screen. Ultrasound pictures will be analysed electronically by means of Photoshop 23.4.2 (Adobe Systems Inc., San Jose, CA, USA). The processed data files of one investigation series will be illustrated in the very same scale on personal computer display.

By using the moving tool from the Photoshop 23.4.2 software, the selected area will be moved to muscle and connective tissue around the nerve to the following positions: 3, 6, 9 and 12 o´clock. The above-described software-based measurements will be performed also for these selections.

Therefore 5 measurements (1 measurement for the nerve and 4 measurements for the surrounding tissue at 3, 6, 9 and 12 o´clock positions) will be performed in each volunteer and the grey scale difference nerve / surrounding tissue with be evaluated according to the following formula:

$$Relative Visibility of the Nerve \left( \% \right)= Grey Scale Value Ulnar Nerve/\frac{Grey Scale Values 0,3,6,9 o´clock}{4}$$

In addition to the graphical, software supported analysis, the Vienna Scoring System ^[[1]](#footnote-1),^^[[2]](#footnote-2)^ for descriptive evaluation of peripheral nerves will be used to mark differences of echotexture:

1. Internal Structure of the nerve visualized
2. The nerve is visualized as a circular or oval-shaped blight halo (epineurium)
3. The nerve is visualized as reflection determined by the anatomy of the surrounded tissue
4. The anatomical position of the nerve shows no response to the ultrasound beam (isoechoic behaviour)

## Pharmacokinetic Analysis

Blood samples: A 4-ml sample of blood (in Lithium-Heparin syringes) will be drawn from an antecubital vein for the determination of plasma ropivacaine concentrations at baseline and 20, 40, 60, 120, 240 and 360 min after performance of nerve blockade (7 blood samples). Blood samples will be kept on ice for a maximum of 60 min before being centrifuged at 4°C and 2600 g for 10 min to obtain plasma. Plasma sampling time points were chosen to capture C_max_ (previously described at t_max_ of 0.44 hours after local block) and terminal half-life (previously described as 2.12h).

The collected samples will be stored at minus 20°C for subsequent analysis. Ropivacaine concentrations in venous plasma will be determined by gas-chromatography/mass spectrometry (GC-MS). Briefly, 100 µL of plasma will be mixed with 100 µL internal standard solution (1,25 µg bupivacaine/mL phosphate buffer pH 7.4) and extracted with 700 µL of n-butyl chloride. The sample will be vortexed gently for 1 min, centrifuged and the supernatant will be transferred into a GC-MS vial.

The GC-MS system consists of a 7890B gas chromatograph coupled with a 7000C GS-MS/MS Triple Quad mass selective detector spectrometer (Agilent, Santa Clara, CA, USA). An autosampler G4513A will be used for splitless injection onto an Agilent J&W HP GC Column (30 m, 0.25 mm, 0.25 µm). The injection volume will be 2µL. Evaluation will be performed with Mass Hunter MS Quantitative Analysis software (Agilent, Santa Clara, CA, USA).

Pharmacokinetic data will be calculated and fitted using a commercially available computer program (Certara Phoenix PK Software). The following PK parameters will be determined: maximum concentration (Cmax), time to maximum concentration (tmax), terminal elimination half- life (t1⁄2), area under the concentration–time curve (AUC) for the sampling period of 6 h (AUC0-6), and the total AUC (AUC0-∞). The AUC for plasma and interstitium will be calculated from non-fitted data by employing the trapezoidal rule. When concentrations are below the lower detection limit for some of the time points, the AUC0-6 will be based on the area under the measurable concentrations. For plasma, apparent total body clearance (CL) and apparent volume of distribution (Vd) are also calculated.

## Objectives of the study

### Primary Outcome Parameter

To evaluate the difference between male and female study subjects in sensory block duration of the ulnar nerve.

### Secondary Outcome Parameters

To evaluate the differences in PK, PD (sensory and motor onset times, duration of motor blockade) and morphometric ulnar nerve characteristics between male and female study subjects.

## Null-hypothesis and alternative-hypothesis

Null-hypothesis: biological gender (male or women) does not influence the primary outcome parameter

Alterative-hypotheses: biological gender (male or women) influences the primary outcome parameter

## Study design

Single centre

Main activities during study period:

- Ulnar nerve blockade
- Morphometric measurements of the ulnar nerve via ultrasound
- Evaluation of sensory and motor blockade
- Blood samples for PKs
- Evaluation of ulnar nerve visibility in ultrasound

# Study population

Potential study participants are selected from a subject database of the Department of Clinical Pharmacology according to the inclusion criteria and asked over the phone whether they are basically interested in participating in such a study. If the potential study participants are interested in principle, appointments for an initial interview will then be made via the study secretariat, during which it can then be clarified whether the subject is suitable for inclusion in the study and whether he or she is willing to take part in the present study. The subject is then informed about the procedure, the risks, the compensation for the study and legal aspects (insurance cover, etc.). During this appointment written informed consent is obtained.

## Inclusion criteria for healthy volunteers

- Healthy male and female volunteers
- Healthy, according to the medical history, ECG, vital signs, laboratory results and physical examination as determined by the Investigator/Sub-Investigator
- Signed written informed consent prior to inclusion in the study
- 18-55 years old inclusive
- BMI: 18 to 35 kg/m^2^
- Ability to understand the full nature and purpose of the study, including possible risks and side effects
- Ability to co-operate with the investigator and to comply with the requirements of the entire study
- Availability to volunteer for the entire study duration and willing to adhere to all protocol requirements

## Exclusion criteria for healthy volunteers

- Any clinically relevant abnormalities at ECG (12 leads)
- Any clinically relevant abnormal physical findings
- Any clinically relevant abnormal laboratory values indicative of physical illness
- Ascertained or presumptive hypersensitivity to the active principle and/or formulations ingredients of the study drug
- History of anaphylaxis to drugs or allergic reactions in general, which the investigator considers may affect the outcome of the study
- Relevant history of malignancy, of renal, hepatic, cardiovascular, respiratory, gastrointestinal, musculoskeletal, skin (particularly at the site of drug application), haematological, endocrine or neurological diseases that may interfere with the aim of the study
- Pregnancy
- Any psychiatric illnesses
- Any anticoagulatory or pain-therapy-related drugs (e.g. NSAIDs, opioids) from one week before until the end of the study
- Using other medications during 1 week before the start of IMP application including OTC
- Participation in another clinical study investigating another IMP within 1 month prior to screening
- Blood donations during 4 weeks prior to this study
- History of drug or alcohol abuse
- Other objections to study participation in the opinion of the investigator

## Reason for withdrawal

- At their own request
- Unreliability and/or lack of cooperation
- Signs of clinically relevant illness that might hamper the performance of the study according to the protocol or the evaluation of safety
- Alcohol or drug abuse
- Occurrence of adverse events (signs and symptoms) or inter-current diseases which the investigator deems to be unacceptable
- Other objections to participate in the study in the opinion of the investigator

# Study drug

## Ropivacaine

Ropivacaine is an amino-amide sodium channel blocker and labelled for nerve blockade and neuraxial anaesthesia.

Ropivacaine is labelled for surgical anaesthesia and acute pain management for the following indications:

- Peripheral nerve blockade
- Surgical infiltration
- Epidural block for surgery including Caesarean section

### Pharmacokinetic data

Formula: C_17_H_26_N_2_O
Chemical name: (S)-1-Propyl-2',6'-dimethyl-2-piperidylcarboxyanilid
Molecular mass: 274.408 g·mol^−1^
Protein binding: 94% (mainly to α_1_-acid glycoprotein)
Metabolism: Hepatic (CYP1A2-mediated)
Elimination half-life: 1.6 - 6 hours (dependent on administration route)
Excretion: Renal (86 %)

### Complete description of indications, contraindications, undesirable and general side effects

See Appendix 1

# Study protocol

Upon signature of the informed consent form and provided that the inclusion/exclusion criteria are fulfilled, subjects will be enrolled in the study.

Overall duration of the study for each individual subject is planned to be two weeks.

## Screening visit (day -14 to day-1)

Prior to inclusion subjects will be informed about the objective and the procedures of the study and about the risks involved. In- and exclusion-criteria, demographic data including underlying disease will be assessed. Each enrolled healthy volunteer will be scheduled to undergo a general physical examination including anamnesis, determination of BMI, drawing blood for laboratory screening tests (including complete blood count, blood coagulation tests, serum electrolytes, creatinine, γ-GT, AST, ALT, CK, total protein, total bilirubin and virology (HBsAg, HBcAg, HCV, HIV-will be accepted if not older than 6 months), Covid-19 PCR test (optional- according to currently valid rules of access to the hospital and/or national regulations), vital signs recording (blood pressure (BP) and heart rate (HR) after 5 minutes rest in supine position) and electrocardiogram (ECG). In addition, urine analyses will be performed. For female participants, an urine pregnancy rapid test will be performed.

## Study day

In the morning of the study day volunteers will be admitted to the clinical research ward which they will not leave until return of sensory and motor function (definitions see below).

The volunteers will receive a plastic cannula (Venflon®) with a switch-valve inserted into an antecubital vein for iv. administration of emergency drugs (in case of hypotension or bradycardia). Thereafter the nerve blocks will be performed as described elsewhere. Each volunteer will receive one ultrasound guided ulnar nerve block on the non-dominant arm.

The sensory evaluation of the nerve block will be assessed with Pinprick testing. The motor evaluation of the nerve block will be assessed by adduction of the thumb.

## Final examination

The final visit (evaluation of ulnar nerve function and puncture site of the nerve block) will be performed up to 7 days after the study via a telephone interview. The volunteers will be called up to study centre in cases of abnormalities.

# METHODS

## Nerve blocks

All ulnar nerve blocks will be performed with ultrasound guidance and a high frequent linear ultrasound probe (8-15 MHz). A transportable ultrasound machine will be used (SonoSite™ X-Port, Bothell, WA, USA). Sterile conditions will be achieved by sterile preparation of the nerve block puncture area and the use of a sterile ultrasound probe cover. Three ml ropivacaine 7.5 mg/ml (= 22.5 mg) for a single peripheral nerve block will provide an effective and selective nerve block.

## Sensory assessment of blockade

The sensory evaluation of the nerve blocks will be assessed before and after blockade with Pinprick Testing (time intervals see below).

## Motor assessment of blockade

Evaluation of motor blockade of the nerve blocks will be assessed before and after blockade according to a four-point scoring system (time intervals see below).

## Definition of onset times

### Sensory blockade

Time from performance of blockade (injection of LA with or without dexamethasone) to Pinprick = 0 in 4 from 5 predefined sensory areas of the ulnar nerve.

### Motor blockade

Time from performance of blockade (injection of LA with or without dexamethasone) to motor score = 0

## Definition of duration of blockade

### Sensory blockade

Time from Pinprick = 0 to Pinprick = 80

### Motor blockade

Time from motor score = 0 to motor score = 1

## Time points

Baseline, 2, 4, 6, 8, 10, 15, 20, 30, 60 min after the block, and then every 30 min until complete recovery.

## Blinding

-

## Power Analysis

A previous study with ropivacaine 0.75% for ulnar nerve blockade showed a duration of sensory blockade of 350 min with the largest SD of 54 minutes.^2^ We expect a true difference of approximately 35 minutes (10%). Assuming a true difference in means between the test and the reference group of 35 minutes, a pooled standard deviation of 54 minutes, the study would require a sample size of 38 for each group (i.e. a total sample size of 76, assuming equal group sizes), to achieve a power of 80% and a level of significance of 5%, for declaring that the duration of block in one biological gender group is longer than in the other biological gender group at a 5 minutes margin of superiority (assuming that a longer duration is desirable). For nonparametric analysis, the sample size is increased by 15% to 90 subjects.

After 2 x 12 cases, we perform an interim analysis for re-evaluation of the initially calculated number of 90 cases (power = 80%, level of significance = 5%). Based on the results of the primary outcome parameter after interim-analysis, a power analysis (to achieve a power of 80% and a level of significance of 5%) to find a nominal difference of 10 to 15% will be performed to determine the number of additional study cases which will be investigated.

## Statistical Analysis

Pharmacodynamic data analysis: The primary outcome is duration of sensory block. Secondary outcomes include onset of sensory block, onset and duration of motor block.
Data will be presented as mean (SD), median [interquartiles] and count (%) and analysed using unpaired two-sided Student *t-*, Mann-Whitney *U-*, log-rank and Fisher exact statistics. Mixed effects linear models will be used to adjust for subject characteristics.

Pharmacokinetic data analysis: Secondary outcomes, including parameters such as C_max_, t_max_and AUC will be estimated and analysed using statistics as listed above.

Morphometric data analysis: Differences between grey scales of nerves and nerve visibility (differences between nerves and surrounding tissue) and differences in the Vienna Scoring System will be compared using unpaired two-sided Student *t-* or Mann-Whitney *U-* statistics as appropriate. Mixed effects linear models will be performed to determine the correlation of nerve visibility with morphometric variables.

# Drug Storage

All study drugs will be stored at room-temperature (20°C-25°C), protected from light, at the Department of Clinical Pharmacology and will be protected against unauthorized access.

Records of the investigational product's delivery to the trial site, the inventory at the site, the use by each subject, and the disposition of unused product will be kept by an experienced research nurse. These records will include dates, quantities, batch/serial numbers, expiration dates, and the unique code numbers assigned to the investigational product and trial subjects.

# Labelling

## Subject identification

Subject numbers 001 - 090 will be used in a consecutive order. Subjects withdrawn from the study will retain their identification code. New replacement subjects enrolled will be assigned a number equal to the study number of the withdrawn subject plus 100 units: e.g. subject 001 will be replaced by the subject 101, subject 02 by subject 102 Sample identification

Each sample will be clearly and unequivocally identified with a label resistant to the storage temperature and containing the following information:

Abbreviated name of the study (Gender_PNB)

Subject number (001-090)

Scheduled time of sampling (hh:min)

Content (P1 / P2).

# Life style restrictions

The following restrictions will apply during the study:

- Intake of concomitant medication except in case of emergency or treatment of AEs
- Intake of excessive alcohol

# Documentation

The following documents must be available in the Investigator’s study file prior to the enrolment of any subject:

- Copy of the positive vote from the Ethics Committee;
- A list of members of the Ethics Committee in accordance with local regulations;
- Notification to local and national regulatory authority;
- Sample of the consent form and subject information to be used;

Data of screening examination will first be kept in separate subject files (source data files) and will be entered into the CRF only if the subject is eligible for trial participation and the data were verified by an investigator.

The subjects will be monitored through the course of the trial and all findings will be recorded.

The records should include the study number, the taking of consent, visit dates of the patient, medical history or examinations administered, laboratory results, concomitant treatment and sample collection times for diagnostic as well as pharmacokinetic samples, any adverse events encountered, and other notes as appropriate.

AEs as reported by the patients will be recorded on separate forms as source data in German or English language and will be transcribed into the CRF after translation in English language. A physician will do the judgement according to the criteria of the ”Adverse Events” page.

Data of medical measurement without print-outs (vital signs, time of trial activities, i.e. time of sampling, administration) performed during the trial will be recorded on worksheets and will be handled as source data. Clinical laboratory parameters will be provided in laboratory print-outs which were signed and dated by the investigator. Comments on all clinically significant abnormal values should be given by the investigator. Data which are subject to the data protection law (i.e. name, address) will not be entered into the CRF.

For error corrections in source data the investigator or an authorized clinical staff member will cross out the part to be corrected (no correction fluid!) with one line, so that the incorrect data is still readable, and then entering the correct data, initialling and dating the change.

All correspondence (e.g. with Ethics Committee) relating to this clinical trial should be kept in appropriate file folders. Records of subjects, source documents, drug inventory sheet and Informed Consents pertaining to the trial must be kept on the Investigator’s File. Records must be retained according to guidelines governing the conduct of the trial.

According to the European Community record retention is required for 15 years.

If the investigator moves, withdraws from an investigation or retires, the responsibility for maintaining the records may be transferred to another person who will accept the responsibility.

# Adverse events

## Definition

An AE is any untoward medical occurrence in a subject or clinical investigation subject that does not necessarily have a causal relationship with any treatment. An adverse event may consist of any medical change (illness, condition, sign or symptom) in a subject after exposure to the study drug and includes:

- an event not seen prior to study drug administration
- an exacerbation of a pre-existing illness, condition, sign or symptom (e.g. increase in asthma attacks)
- a recurrence of an intermittent illness, condition, sign or symptom (e.g. migraine)
- an illness or condition diagnosed after study drug administration even though it may have been present prior to administration (e.g. cancer)

An adverse event does not include:

- a continuous persistent illness, condition, sign or symptom present before study drug administration which does not unexpectedly progress or change in severity following study drug administration
- the disease being studied or signs or symptoms associated with the disease (e.g., pain and swelling due to osteoarthritis of the knee)

## Complications of Ultrasound Guided Peripheral Nerve Blocks

Nerve block: Overall, the complication rate of ultrasound guided peripheral nerve blocks is low. The main complication is intraneuronal puncture, which leads in the minority of cases to prolonged paraesthesia or nerve damage (incidence unknown). Due to the fact that ultrasound is used for single peripheral nerve blockade, intraneuronal (intraepineural) needle tip position and subsequent administration of LA (intraepineurally) can be excluded. Haematoma formation is another possible complication, but the nerve will be blocked at areas (see above) without adjacent vessels. Infection is also a possible complication, but sterile conditions are used for the procedures.

Local anaesthetic: Ropivacaine is used as LA, which shows the lowest potency of systemic side effects. Due to the low dose of ropivacaine used in the present study (3x7.5mg in 3ml = 22.5mg = 0.75%) any systemic side effects (bradycardia, hypotension, central neurotoxic effects such as convulsion, etc.) can be excluded. Nevertheless, the volunteers are haemodynamically monitored throughout the study period for possible emergency intervention.

## Documentation and Reporting of Adverse Events

AEs will be documented throughout the entire study. In addition, SAEs which are reported to the investigator within 30 days after study termination have also to be documented using the SAE reporting form.

## Serious adverse events (SAEs)

A serious adverse event is an adverse event that:

- results in death, or
- is life-threatening (the subject was at risk of death at the time of the event)
- requires in-patient hospitalisation or prolongation of existing hospitalisation
- results is persistent or significant disability / incapacity, or
- is a congenital anomaly / birth defect, or
- is another medically important condition that may be considered a SAE when, based upon appropriate medical judgment, it may jeopardize the subject or may require intervention to prevent one of the outcomes listed above

## Causality Assessment between Adverse Events and study medication

A causality assessment between adverse events and study medication will be performed as follows:

**Not related -** clearly unrelated to the use of the test product; an evident alternative cause must be available.

**Possible -** causal relationship to the test product appears unlikely, but cannot be ruled out with reasonable certainty.

**Probable -** felt to be causally related to the test product with a high degree of likelihood.

## Severity of the adverse event

The severity of AEs will be graded according to the following scale:

**Mild** - the adverse event does not interfere in a significant manner with the subject’s normal functioning.

**Moderate -** the adverse event produces some impairment of functioning but is not hazardous to health.

**Severe -** the adverse event produces significant impairment of functioning or incapacitation and is a definite hazard to the subject’s health.

## Information of the regulatory authorities and the IRB-IEC

The sponsor has to inform the involved regulatory authorities and the investigator has to inform the involved ethics committee about each serious or unexpected AE/adverse drug reaction (that is an experience not previously reported - in nature, severity or incidence - in the current Summary of Product Characteristics that in the opinion of the investigator or sponsor might jeopardize the safety of the subjects or the further conduct of the study).

# Premature termination of the trial

The trial will be terminated prematurely in any of the following cases for example:

- If adverse events occur which are so serious that the resulting risk-benefit ratio becomes unacceptable
- If the number of drop-outs is so large that proper conclusion of the trial is no longer a realistic possibility
- If the results of parallel clinical trials reveal unacceptable risks
- If results of any interim analyses and the status of drug development should change, such that the trial would no longer be a necessary part of the clinical program
- Attempted or proven fraud
- Slow recruitment
- Poor quality of data
- Non-compliance with protocol.

# REMOVAL OF SUBJECT FROM TRIAL

Any subject who discontinues participation to the trial within two hours after onset of drug administration, is defined as ”drop-out”. Although subjects are not obliged to give reason(s) for withdrawal, the investigator should make a reasonable effort to ascertain the reason(s), while fully respecting the subject’s rights.

The Investigator may remove the subject from the trial for any of the following reasons:

- Failure to observe the trial conditions or instructions from the trial team
- Failure to comply with any aspect of the protocol
- Occurrence of AEs
- Recurrent diseases which the investigator deems to be unacceptable
- Detection of drugs or addictive substances

For follow-up procedures of AEs the subjects will stay under medical supervision (in hospital or via telephone contact) unless the physician believes that all adverse events have resolved or a final assessment can be made. Appropriate control measurements may be initiated for safety parameters. All AEs will be documented and assessed by physicians. Serious adverse events will be communicated to the responsible authorities within the legal timelines.

If a subject is removed or discontinues, a replacement subject will be enrolled who will follow the same procedure as described for the original subject. The reason(s) for drop-out must be recorded.

# STATISTICAL / ANALYSIS SECTION

## Sample size calculation

A previous study with ropivacaine 0.75% for ulnar nerve blockade showed a duration of sensory blockade of 350 min with the largest SD of 54 minutes.^2^ Assuming a nominal difference in means between the study groups of 35 minutes, a pooled standard deviation of 54 minutes, the study would require a sample size of 39 for each group (i.e. a total sample size of 78, assuming equal group sizes), to achieve a power of 80% and a level of significance of 5%. To enable nonparametric analysis, the sample size is increased by 15% to 90 subjects. For a larger nominal 15% difference, the total sample size is reduced to 42 subjects.

## Interim-Analysis

After 2 x 12 cases, we perform an interim analysis for re-evaluation of the initially calculated number of 90 cases (power = 80%, level of significance = 5%). Based on the results of the primary outcome parameter after interim-analysis, a power analysis (to achieve a power of 80% and a level of significance of 5%) to find a nominal difference of 10 to 15% will be performed to determine the number of additional study cases which will be investigated.

## Randomization

-

## PD, PK and morphometric data analysis

Pharmacodynamic data analysis: The primary outcome is duration of sensory block. Secondary outcomes include onset of sensory block, onset and duration of motor block. Data will be presented as mean (SD), median [interquartiles] and count (%) and analysed using unpaired two-sided Student *t-*, Mann-Whitney *U-*, log-rank and Fisher exact statistics. Mixed effects linear models will be used to adjust for subject characteristics. Data will be analysed using Stata 17.0 and NCSS 2020.

Pharmacokinetic data analysis: Secondary outcomes, including parameters such as C_max_, t_max_and AUC will be estimated and analysed using statistics as listed above.

Morphometric data analysis: Differences between grey scales of nerves and nerve visibility (differences between nerves and surrounding tissue) and differences in the Vienna Scoring System will be compared using unpaired two-sided Student *t-* or Mann-Whitney *U-* statistics as appropriate. Mixed effects linear models will be performed to determine the correlation of nerve visibility with morphometric variables.

# ETHICAL SECTION

## Safety precautions

During the period of study visits subjects will be observed by a physician or an experienced nurse.

## Inconveniences and risks for subjects

Side effects of *ropivacaine* (as enlisted in detail in the Summary of Product Characteristics, Appendix 1) include local numbness, dizziness, hypotension, nausea, vomiting, respectively.

## Risk/benefit assessment

Overall, the risks associated with the study are low.

### Individual benefit

Subjects do not have direct a health benefit from participation. The benefit is attributed to overall benefits for patients and scientific community.

## Adverse events

An AE is any untoward medical occurrence in a subject or clinical investigation subject that does not necessarily have a causal relationship with any treatment. An adverse event may consist of any medical change (illness, condition, sign or symptom) in a subject after exposure to the study drug and includes:

- an event not seen prior to study drug administration
- an exacerbation of a pre-existing illness, condition, sign or symptom (e.g. increase in asthma attacks)
- a recurrence of an intermittent illness, condition, sign or symptom (e.g. migraine)
- an illness or condition diagnosed after study drug administration even though it may have been present prior to administration (e.g. cancer)

An adverse event does not include:

- a continuous persistent illness, condition, sign or symptom present before study drug administration which does not unexpectedly progress or change in severity following study drug administration
- the disease being studied or signs or symptoms associated with the disease (e.g., pain and swelling due to osteoarthritis of the knee)

AEs will be documented throughout the entire study. In addition, SAEs which are reported to the investigator within 30 days after study termination have also to be documented using the SAE reporting form.

## Serious adverse events

A serious adverse event is an AE that:

- results in death, or
- is life-threatening (the subject was at risk of death at the time of the event)
- requires in-patient hospitalisation or prolongation of existing hospitalisation
- results in persistent or significant disability / incapacity, or
- is a congenital anomaly / birth defect, or
- is another medically important condition that may be considered a SAE when, based upon appropriate medical judgment, it may jeopardize the subject or may require intervention to prevent one of the outcomes listed above

A causality assessment between adverse events and study medication will be performed as follows:

*Not related* - clearly unrelated to the use of the test product; an evident alternative cause must be available.

*Possible* - causal relationship to the test product appears unlikely, but cannot be ruled out with reasonable certainty.

*Probable* - felt to be causally related to the test product with a high degree of likelihood.

The severity of AEs will be graded according to the following scale:

*Mild* - the adverse event does not interfere in a significant manner with the subject’s normal functioning.

*Moderate* - the adverse event produces some impairment of functioning but is not hazardous to health.

*Severe* - the adverse event produces significant impairment of functioning or incapacitation and is a definite hazard to the subject’s health.

The sponsor has to inform the involved regulatory authorities and the investigator has to inform the involved ethics committee about each serious or unexpected AE/adverse drug reaction (SUSAR, that is an experience not previously reported - in nature, severity or incidence - in the current Summary of Product Characteristics that in the opinion of the investigator or sponsor might jeopardize the safety of the subjects or the further conduct of the study). SUSARs not fatal or not life threatening will be reported to the IRB-IEC and the competent regulatory authorities within 15 calendar days. SUSARs that lead to death or are life threatening will be reported within 7 calendar days.

## Insurance

For all study participants insurance is provided. Insurance is covered at the Zürich Insurance Group, Leopold-Ungar-Platz 2, A-1190 Vienna, with the policy number 07229622-2 and is valid from 01.11.2022 till 01.11.2024, 00.00 o’clock respectively. By request the study participants can examine the insurance papers.

# Ethical and legal aspects

The study will be performed in accordance with the Declaration of Helsinki (1964), including current revisions, the Austrian Drug Law (Arzneimittelgesetz, AMG) (2004), Good Clinical Practice (GCP) guidelines of the European Commission and the Good scientific practice guidelines of the Medical University of Vienna. Approval from the local ethics committee will be sought before initiating the study. All subjects enrolled at the Department of Clinical Pharmacology will be insured through the Department of Clinical Pharmacology in accordance with §38 of the Austrian Medicines Act.

# Audit and Inspection

Upon request, the Investigator will make all study-related source data and records available to a qualified quality assurance auditor mandated by the competent authority inspectors. The main purposes of an audit or inspection are to confirm that the rights and welfare of the subjects have been adequately protected, and that all data relevant for assessment of safety and efficacy of the investigational product have appropriately been reported to the sponsor.

# ADMINISTRATIVE SECTION

## Publication of study results

Publication of study results from this investigation in an appropriate medical journal will be anticipated. Before submission the manuscript will be circulated to all parties involved.

## Protocol amendment

If any modifications become necessary or desirable, these will be documented in writing; major changes require the approval of all investigators and the local ethics committee and competent authorities.

## Monitoring

The CRFs will be reviewed by the monitor. Also source data verification will be performed by the monitor to ensure accurate, consistent, reliable and complete data. Drug storage will be checked by the monitor. The study products should be dispensed by the investigator, or by a qualified individual under the investigator’s supervision.

# FUNDING

The Department of Clinical Pharmacology of the Medical University of Vienna will serve as sponsor for this study with research funds. No external funding will be used for this study and this is not an industrial driven trial.

# REFERENCES

1. Marhofer P, Pilz-Lubsczyk B, Lönnqvist PA, Fleischmann E. Ultrasound-guided peripheral regional anaesthesia: a feasibility study in obese versus normal-weight women. *Int J Obes (Lond)*. 2014;38 (3):451-455.

2. Marhofer D, Kettner SC, Marhofer P, Pils S, Weber M, Zeitlinger M. Dexmedetomidine as an adjuvant to ropivacaine prolongs peripheral nerve block: a volunteer study. *Br J Anaesth*. 2013;110 (3):438-442.

3. Fourny N, Beauloye C, Bernard M, Horman S, Desrois M, Bertrand L. Sex Differences of the Diabetic Heart. *Front Physiol*. 2021;12 661297.

4. Khan SS, Beach LB, Yancy CW. Sex-Based Differences in Heart Failure: JACC Focus Seminar 7/7. *J Am Coll Cardiol*. 2022;79 (15):1530-1541.

5. Picone DS, Stoneman E, Cremer A et al. Sex Differences in Blood Pressure and Potential Implications for Cardiovascular Risk Management. *Hypertension*. 2022; online ahead of print.

6. Wang SE, Dashti SG, Hodge AM et al. Mechanisms for the Sex-Specific Effect of H. Pylori on Risk of Gastroesophageal Reflux Disease and Barrett’s Esophagus. *Cancer Epidemiol Biomarkers Prev*. 2022;31 (8):1630-1637.

7. Camorcia M, Capogna G, Columb MO. Effect of sex and pregnancy on the potency of intrathecal bupivacaine: determination of ED₅₀ for motor block with the up-down sequential allocation method. *Eur J Anaesthesiol*. 2011;28 (4):240-244.

8. Huang YY, Chang KY. Sensory block level prediction of spinal anaesthesia with 0.5% hyperbaric bupivacaine: a retrospective study. *Sci Rep*. 2021;11 (1):9105.

9. Cheema S, Richardson J, McGurgan P. Factors affecting the spread of bupivacaine in the adult thoracic paravertebral space. *Anaesthesia*. 2003;58 (7):684-687.

10. Gaudreault F, Drolet P, Fallaha M, Varin F. Modeling the anesthetic effect of ropivacaine after a femoral nerve block in orthopedic patients: a population pharmacokinetic-pharmacodynamic analysis. *Anesthesiology*. 2015;122 (5):1010-1020.

11. Tang S, Wang J, Tian Y et al. Sex-dependent prolongation of sciatic nerve blockade in diabetes patients: a prospective cohort study. *Reg Anesth Pain Med*. 2019 ; online ahead of print

12. Cullion K, Petishnok LC, Ji T, Zurakowski D, Kohane DS. The Duration of Nerve Block from Local Anesthetic Formulations in Male and Female Rats. *Pharm Res*. 2019;36 (12):179.

13. Marhofer D, Marhofer P, Kettner SC et al. Magnetic resonance imaging analysis of the spread of local anesthetic solution after ultrasound-guided lateral thoracic paravertebral blockade: a volunteer study. *Anesthesiology*. 2013;118 (5):1106-1112.

14. Marhofer D, Karmakar MK, Marhofer P, Kettner SC, Weber M, Zeitlinger M. Does circumferential spread of local anaesthetic improve the success of peripheral nerve block. *Br J Anaesth*. 2014;113 (1):177-185.

15. Marhofer P, Columb M, Hopkins PM et al. Dexamethasone as an adjuvant for peripheral nerve blockade: a randomised, triple-blinded crossover study in volunteers. *Br J Anaesth*. 2019;122 (4):525-531.

16. Opfermann P, Marhofer P, Hopkins PM et al. Generic versus reference listed ropivacaine for peripheral nerve blockade: A randomised, triple-blinded, crossover, equivalence study in volunteers. *Eur J Anaesthesiol*. 2021;38 (Suppl 2):S113-S120.

17. Marhofer P, Greher M, Kapral S. Ultrasound guidance in regional anaesthesia. *Br J Anaesth*. 2005;94 (1):7-17.

18. Walter U, Tsiberidou P. Differential age-, gender-, and side-dependency of vagus, spinal accessory, and phrenic nerve calibers detected with precise ultrasonography measures. *Muscle Nerve*. 2019;59 (4):486-491.

19. Fujimaki Y, Kuwabara S, Sato Y et al. The effects of age, gender, and body mass index on amplitude of sensory nerve action potentials: multivariate analyses. *Clin Neurophysiol*. 2009;120 (9):1683-1686.

20. Keplinger M, Marhofer P, Moriggl B, Zeitlinger M, Muehleder-Matterey S, Marhofer D. Cutaneous innervation of the hand: clinical testing in volunteers shows high intra- and inter-individual variability. *Br J Anaesth*. 2018;120 (4):836-845.

21. Eichenberger U, Stöckli S, Marhofer P et al. Minimal local anesthetic volume for peripheral nerve block: a new ultrasound-guided, nerve dimension-based method. *Reg Anesth Pain Med*. 2009;34 (3):242-246.

22. Hermanns H, Werdehausen R, Hollmann MW, Stevens MF. Assessment of skin temperature during regional anaesthesia-What the anaesthesiologist should know. *Acta Anaesthesiol Scand*. 2018;62 (9):1280-1289.

23. van Haren FG, Kadic L, Driessen JJ. Skin temperature measured by infrared thermography after ultrasound-guided blockade of the sciatic nerve. *Acta Anaesthesiol Scand*. 2013;57 (9):1111-1117.

# Appendices

Appendix 1: Summary of product characteristics ropivacaine 7.5 mg/mL (0.75%)

1. Ultrasound Guidance in Regional Anaesthesia – Principles and practical implementation. Oxford University Press, 2^nd^ Edition, p 209, 2010 by Peter Marhofer, ISBN 978-0-19-960482-1 [↑](#footnote-ref-1)
2. More Notable Names in Anaesthesia, The Choir Press, p 232-3, 2021 by Alistair G McKenzie, ISBN: 978-1-78963-170-8 [↑](#footnote-ref-2)
