# Supplementary material for: The impact of biological sex in peripheral nerve blockade: A prospective pharmacodynamic, pharmacokinetic and morphometric study in volunteers
Source: PLoS One. 2024 Jan 26;19(1):e0297095. doi: 10.1371/journal.pone.0297095 (PMC10817111; doi:10.1371/journal.pone.0297095)
Supplement: Attachment — Submitted filename: Response to Reviewers_Plos One_Sex differences_signed.pdf [file pone.0297095.s003.pdf]

Markus Zadrazil, MD PhD
Medical University of Vienna
Department of Anesthesia, General Intensive Care Medicine and Pain Therapy
Spitalgasse 23, A-1090 Vienna, Austria
markus.zadrazil@meduniwien.ac.at

ORCID iD: 0000-0003-2138-5424

Date: Dec. 11th, 2023

Dear Editor, dear Lalit Gupta!

Thank you very much for considering our study for publication in PLOS ONE.
Please find attached our point-to-point answers to each point raised by the reviewers concerning the manuscript for the research article with the title **"The impact of biological sex in peripheral nerve blockade: A prospective pharmacodynamic, pharmacokinetic and morphometric study in volunteers"**.

Reviewer #1: *Volunteers participated in a clinical trial which aimed to investigate the impact of biological sex on pharmacodynamic, pharmacokinetic and morphometric characteristics for peripheral nerve blockade.*

Minor revisions:
1- Abstract and line 197: Power calculations include significance levels rather than p-values. Replace "P<0.05" at all occurrences.

- Thank you very much for this comment. It is correct in that ''P<0.05'' should be replaced in power calculations and that this is specified better by the term ''alpha error of 5%'', which has been changed throughout as suggested.

2- Abstract: Briefly state the statistical methods from which p-values were estimated.

- Mann-Whitney *U*-statistics were used for the data in the abstract and this has been added as suggested (line 34 and 35).

3- Line 163: State the statistical testing method which achieves 80% power. Perhaps it is the t-test.

- As requested by the reviewer, the unpaired Student t-statistic has been inserted for the power calculation.

4- Lines 182 and 204: Grammatical error: Fisher's exact.

- The grammatical error has been corrected.

5- Line 185: State the underlying covariance structure used in the linear mixed effects models and the criteria for selecting it.

- In the used statistical software Stata, the covariance matrix is defaults to ''Identity'' from others for single-variable random-effects specifications in subjects. This has now been added as suggested for completeness by the reviewer.

6- Table 1: For side, provide the percentage corresponding to the left or right side.

- For side, the percentage for the left side has been added in table 1 (left 83.3%).

7- Line 219: Provide a reference for the mathematical method used to calculate AUC.

- The mathematical method used was the trapezoid rule. The following text was inserted in the manuscript "AUC, calculated according to the trapezoidal rule".

8- Table 3: Include sample sizes in the header row.

- The sample sizes (Women, n=12 and Men, n=12) have been added in the header of table 3.

9- Indicate if any adverse events occurred during the course of the study.

- It has been indicated, that no adverse events occurred during the course of the study – line 232/233.

Reviewer #2: *In the age of personalized medicine all details are important that makes a difference between individuals or interindividual differences can be excluded. This is the first human study investigating the impact of biological sex in peripheral nerve blockade. Regional anesthesia plays an important role not only during the surgery but in the postoperative period as well, and in other areas of medicine, including malignancies and obstetrics. Most of amid local anesthetics (including ropivacaine) are highly cardiotoxic compounds. The knowledge that higher peak serum levels may be expected in females may have importance in long term therapy using epidural catheters.*

- Thank you very much for this remark, appreciating our work.

Journal requirements:

- In addition to the revisions, I sought permission from the study sponsor to publish the study protocol under CC BY 4.0 if our work is accepted. I uploaded the formal statement signed by the study sponsor and the study protocol with the removed confidentiality notice.

Following the executed revisions, we hope our manuscript meets the stringent criteria of the Journal for publication in PLOS ONE.

Sincerely yours,

Markus Zadrazil, MD PhD (on behalf of all Co-Authors)
